# Effects of oxygen vacancies on the structural and optical properties of β-Ga_2_O_3_

**DOI:** 10.1038/srep40160

**Published:** 2017-01-09

**Authors:** Linpeng Dong, Renxu Jia, Bin Xin, Bo Peng, Yuming Zhang

**Affiliations:** 1 Wide Bandgap Semiconductor Technology Disciplines State Key Laboratory, Xidian University, Xi’an 710071, China

## Abstract

The structural, electronic, and optical properties of β-Ga_2_O_3_ with oxygen vacancies are studied by employing first-principles calculations based on density function theory. Based on the defects formation energies, we conclude the oxygen vacancies are most stable in their fully charge states. The electronic structures and optical properties of β-Ga_2_O_3_ are calculated by Generalized Gradient Approximation + U formalisms with the Hubbard U parameters set 7.0 eV and 8.5 eV for Ga and O ions, respectively. The calculated bandgap is 4.92 eV, which is consistent with the experimental value. The static real dielectric constants of the defective structures are increased compared with the intrinsic one, which is attributed to the level caused by the Ga-4s states in the bandgap. Extra peaks are introduced in the absorption spectra, which are related to Ga-4s and O-2p states. Experimentally, β-Ga_2_O_3_ films are deposited under different O_2_ volume percentage with ratio-frequency magnetron sputtering method. The measured results indicate that oxygen vacancies can induce extra emission peaks in the photoluminescence spectrum, the location of these peaks are close to the calculated results. Extra O_2_ can increase the formation energies of oxygen vacancies and thus reduce oxygen vacancies in β-Ga_2_O_3_.

Among the transparent conducting oxides (TCOs), monoclinic β-Ga_2_O_3_ is a promising semiconductor with bandgap of 4.9 eV, excellent chemical and thermal stability has gained considerable attention for many applications^1^,^2^. It has been widely used as photocatalyst, solar-blind UV detectors, gas sensors, and short wavelength light emitting diodes^3^,^4^,^5^,^6^,^7^. Besides, it is a promising candidate for power devices as higher Baliga’s figures of merit (over 3000) and higher breakdown field (8 MV/cm), a lower cost and more easily accessible properties compared with the conventional power semiconductors such as SiC and GaN^8^,^9^,^10^,^11^. However, like the traditional TCOs such as ZnO, pure β-Ga_2_O_3_ usually presents an n-type characteristic, and a p-type doped β-Ga_2_O_3_ is hard to get, which hampers its further applications. As an intrinsic defect, oxygen vacancy are usually supposed to be the reason of the n-type property of the intrinsic TCOs. While some theoretical calculation results suggest that the oxygen vacancies act as deep donors in β-Ga_2_O_3_, which make this issue controversial^12^,^13^,^14^. In various manufacture procedures of β-Ga_2_O_3_, oxygen vacancies can be widely introduced. The ambient conditions, especially the oxygen pressure and post-annealed atmosphere are directly related with the concentration of oxygen vacancies. Oxygen vacancies can introduce extra peaks in the bandgap of β-Ga_2_O_3_, which can affect the efficiency and accuracy of the optical devices such as solar-blind UV detectors. However, taking advantage of the oxygen vacancies, amorphous Ga_2_O_3_ has been used as a resistance random access memory (RRAM)^15^,^16^,^17^. Thus, the oxygen vacancies have a significant impact on the application of β-Ga_2_O_3_.

Recently, first-principles calculations based on density functional theory (DFT) have been used for many studies of the material properties such as optics, magnetism and electronic structures^18^,^19^,^20^,^21^. The theoretical calculation can give a deep insight of the material, which can help us get further acquainted with the material itself. The formation energy of the oxygen vacancies of β-Ga_2_O_3_ has been investigated in the past years, and the results can vary with different functionals and approximation methods^12^,^13^,^14^. Up to now, few studies of the effects on the structural and optical properties caused by oxygen vacancies have been discussed systematically. Moreover, the electronic structures of β-Ga_2_O_3_ with oxygen vacancies have not been reported. While in the application of β-Ga_2_O_3_, the oxygen vacancies can have a great influence on the performance of the devices. Thus, it is necessary to detailedly discuss the oxygen vacancies of β-Ga_2_O_3_.

It is well known that the electronic structures of semiconductors are not well described by generalized gradient approximation (GGA) and local-density approximation (LDA) functionals, which will lead to an underestimation of bandgap in semiconductors^22^,^23^,^24^,^25^. As a result, the defects states caused by the defects are not correctly treated. The accurate electronic structures can be described by more elaborate approaches, such as hybrid Hartree-Fock (HF) density functionals, Heyd-Scuseria-Ernzerhof (HSE) and the screened exchange (sX)^14^,^26^. However, these accurate methods are limited to the computational resources.

In this paper, first-principles based on all-electron DFT is used to study the atomic structures, formation energies, electronic structures and optical properties of the intrinsic β-Ga_2_O_3_ with different oxygen vacancies. In order to get a reasonable result, the GGA+U approach is used, which is computational frugally compared with other hybrid density functionals and also can give an accurate description by controlling the Hubbard U parameter^27^,^28^.

## Results and Discussion

### Structural properties

**Figure 1:**
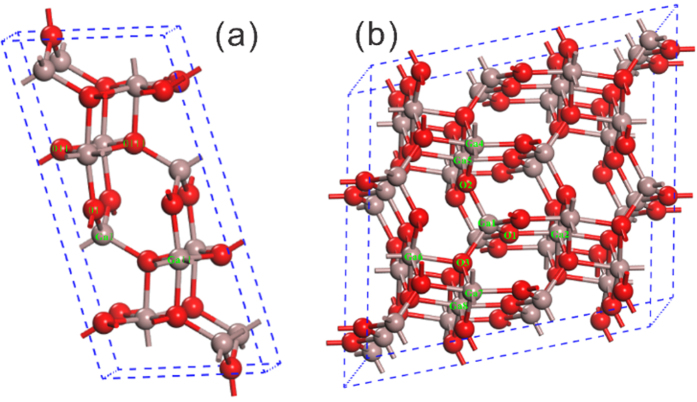
The conventional cell (**a**), and defective supercell (**b**) of β-Ga_2_O_3_. Here, the Ga and O atoms are demonstrated by grown and red spheres, respectively.

Monoclinic structural β-Ga_2_O_3_ with C2/m symmetry can be described with four lattice parameters, i.e, a, b, c and β. Figure 1(a) shows the optimized conventional cell of β-Ga_2_O_3_. The structural parameters of β-Ga_2_O_3_ based on our calculation results and other previous theoretical with experimental results are listed in Table 1. The calculation results are in good agreement with other results derived from different functionals, which indicate our optimization method is reasonable.

There are three types of O sites in β-Ga_2_O_3_ cell as shown in Fig. 1(a). As a result, three O vacancies exist, which are denoted as *V*_OI_, *V*_OII_ (both are 3-fold) and *V*_OIII_ (4-fold), respectively. For *V*_OI_, there are two 6-fold Ga ions and one 4-fold Ga ion surrounded, while two 4-fold Ga ions and one 6-fold Ga ion are adjacent to *V*_OII_. For Ga ions, there are two nonequivalent sites, the 4-fold one and 6-fold one, respectively. The crystalline structures are described in terms of GaO_6_ octahedron and GaO_4_ tetrahedron chains. Based on the former optimized cell, we calculate the defective structures with the 1 × 2 × 2 supercell shown in Fig. 1(b).

### Defects formation energies

The formation energy of an oxygen vacancy D in β-Ga_2_O_3_ with charge state q is given by^30^:


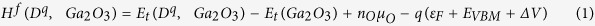


Where 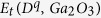 is the total energy of the supercell with an oxygen vacancy *D* in charge state *q*, 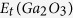 is the total energy of the β-Ga_2_O_3_ perfect supercell structure. *μ*_*O*_ is the chemical potential of O which we use the potential of O_2_ molecule as a reference, *n*_*O*_ denotes the numbers of the vacancies in the supercell. *ε*_*F*_ is the Fermi level measured from the top of the valence band *E*_*VBM*_ and *ΔV* is the average potential difference between the defective supercell and the perfect one.

From Eq. (1), the formation energy of an oxygen vacancy in charge state q can be determined. In this method, the chemical environment difference between the different finite defective supercells, which leads to the errors in the formation energy, are taken into account. Besides, *E*_*VBM*_ varies with the different O vacancies and charge states, which can lead to unreasonable formation energies results. Therefore, it is necessary to align the band structures of the different supercells^31^. *E*_*VBM*_ of the defective supercells is defined by^30^:


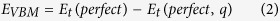


Where 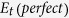 is the total energy of neutral perfect supercell and 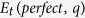 is the total energy of the perfect supercell in charge state q.

The O defects formation energy is associated with oxygen condition in the synthesis process of β-Ga_2_O_3_, and the oxygen chemical potential varies with the different ambient atmospheres. Both *μ*_*O*_ and 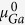 are confined by the phase equilibrium condition of β-Ga_2_O_3_. The range of *μ*_*O*_ is between the oxygen poor (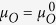, where 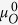 is the total energies per atom of molecular O_2_) and oxygen rich (
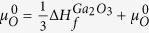
, where 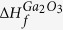 denotes the formation energy of β-Ga_2_O_3_. 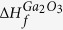 is derived from 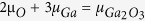, where 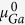 is the total energy per atom of metallic Ga).


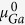
 and 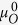 are obtained by separate calculations of total energies of metallic Ga with orthorhombic structure and molecular O_2_, respectively. For O_2_, an isolated O_2_ molecule is placed in a cubic cell with the dimension of 15 × 15 × 15 Å. In this case, only the Γ-point is used. The O-O bond length is 1.24 Å, which is in good agreement with experiment value^32^. For the potential of Ga, an orthorhombic structure is used. The formation energy of β-Ga_2_O_3_ in our calculation results is 3.19 eV per O atom.

**Figure 2:**
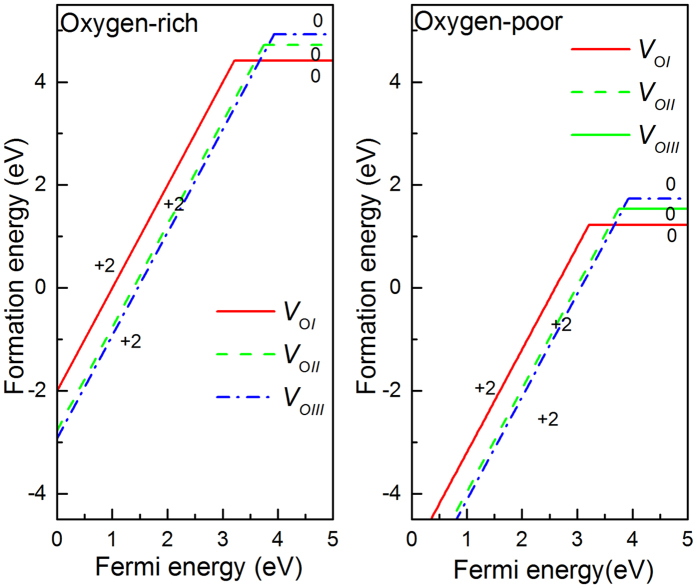
Defects formation energies as a function of the Fermi energy under the oxygen-rich and oxygen-poor for different oxygen vacancies charge states.

The formation energies for different O vacancies with different charge states as a function of the Fermi energy are obtained by the GGA+U method, and the results are shown in Fig. 2. Both oxygen-rich and oxygen-poor cases are calculated. For each defect, only the charge state in the most energetically favorable at a given Fermi energy is shown. The defects formation energies vary with the Fermi level *ε*_*F*_. When *ε*_*F*_ is close to valence band, the stable charge states correspond to 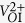, 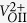 and 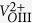, respectively. With the *ε*_*F*_ moving up, the neutral defects are dominant. There are no +1 charge state of oxygen vacancies, which indicates that +1 charge state is not stable for all three type vacancies. The followed discussion we will focus only on these stable structures, namely, neutral and +2 charge states.

Thermodynamic transitions between the different charge states of the same defect are denoted by the kinks as shown in Fig. 2, which are derived from the formation energies. Measured from the valence band maximum (VBM), the ε(+2/0) transition level of 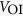, 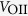 and 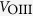 are 3.2 eV, 3.7 eV and 3.9 eV, respectively. The deep transition levels mean all the oxygen vacancies act as deep donors. When Fermi level *ε*_*F*_ locates at the mid-gap around, the charged vacancies are more stable than the neutral ones. Under oxygen-poor atmosphere, the negative oxygen vacancies formation energy make the vacancies easy to form. While in the case of oxygen-rich, the positive formation energy of neutral oxygen vacancies is higher than the ones under oxygen-poor atmosphere. As a result, it is hard to generate a high concentration of oxygen vacancies under this atmosphere. These results indicate the oxygen vacancies are hard to be effective n-type donors, and under oxygen-poor atmosphere, the high compensation effects between oxygen vacancies and p-type acceptor impurity may be the reason of the difficulty to get a well performance p-type β-Ga_2_O_3_. Our conclusions are consistent with other previous theoretical studies^13^,^14^.

### Electronic structures

**Figure 3:**
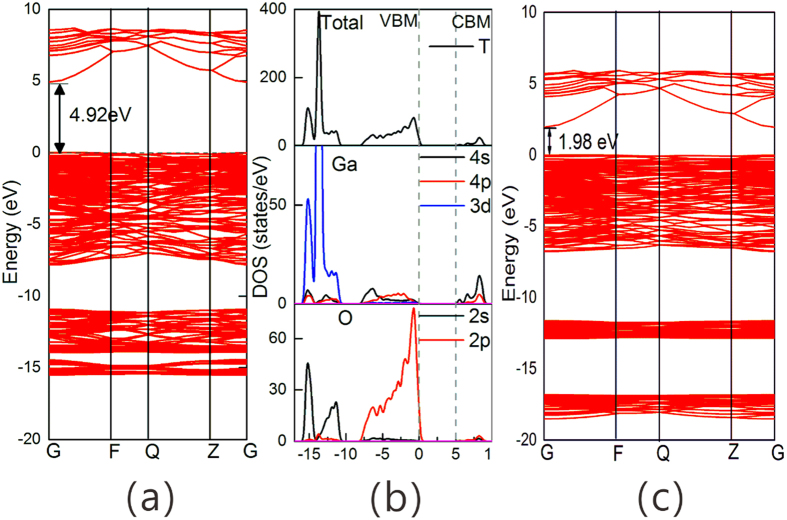
Band structure (a), DOS (b) under GGA+U, and band structure under GGA of intrinsic β-Ga_2_O_3_.

The calculated band structure and densities of states (DOS) of the intrinsic β-Ga_2_O_3_ under GGA+U approximation are shown in Fig. 3(a,b). The bandgap of β-Ga_2_O_3_ in our calculation result is about 4.92 eV and both the VBM and the conduction band minimum (CBM) are situated at the G point, which means the material belongs to the direct bandgap semiconductor. These calculated results are nearly consistent with the results in previous experiment^2^. To illustrate the effects of the Hubbard U, the band structure of β-Ga_2_O_3_ under GGA approximation is also shown in Fig. 3(c). The bandgap of β-Ga_2_O_3_ under GGA approximation is only 1.98 eV, which is far deviated from the value of experimental result. The band structure of β-Ga_2_O_3_ shows a much flat valence band, indicating a large hole effective mass and leading to the low mobility of hole, which hamper the fabrication of p-type β-Ga_2_O_3_. From the DOS results, the valence band of the intrinsic β-Ga_2_O_3_ is composed of three subbands. The upper subband consists mainly of O 2p states with a width of about 7.8 eV. The middle subband is formed mainly by Ga 3d states at about 10.5 eV below the VBM. The lowest one consists mainly of O 2 s along with Ga 3d states and locates at 14.8 eV below the VBM. These bandgap, VB and CB features of β-Ga_2_O_3_ are consistent with previous theoretical results, which indicate our GGA+U method is valid^18^.

**Figure 4:**
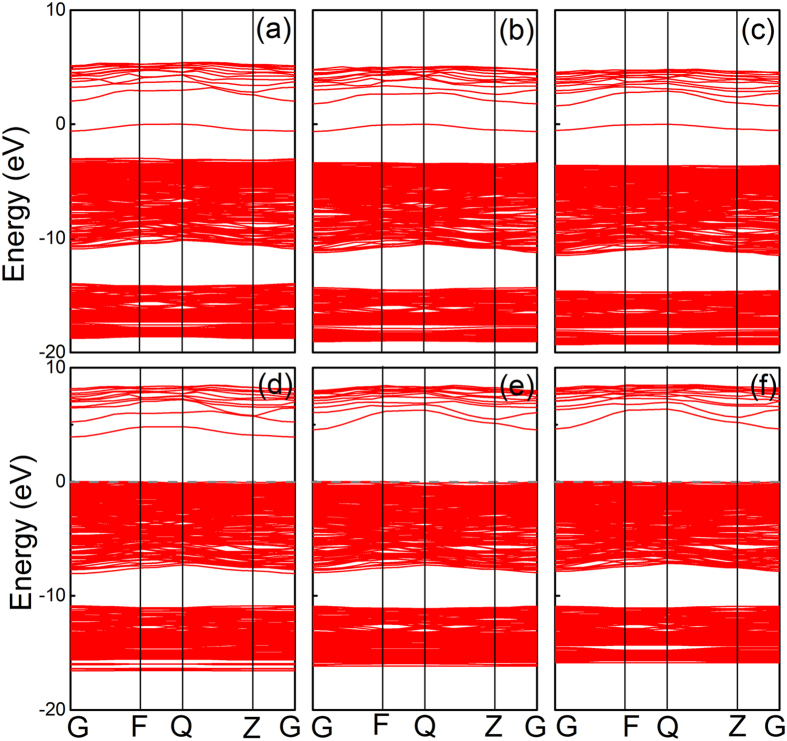
Band structures of β-Ga_2_O_3_ with oxygen vacancies in (**a**) 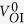, (**b**) 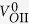, (**c**) 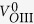, (**d**) 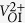, (**e**) 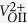, and (**f**) 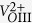 states.

When an oxygen ion is removed, a defective β-Ga_2_O_3_ model with oxygen vacancy is created. For the neutral oxygen vacancies (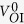, 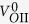 and 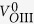), a defective level occupied by two electrons appears, which are shown in Fig. 4. The total energy of the defective β-Ga_2_O_3_ is lowered by the attraction of the surrounded Ga ions, and these ions sites distortion leave the defects level set at the middle of the bandgap. When the vacancies carry with two positive charges (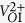, 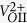 and 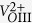), the results are very different from the neutral ones. The defects levels are unoccupied, the outward of surrounded Ga ions make the Ga-O bond strengthen, leading to the decrease of the formation energy. As a result, the defective level moves to the conduction band. These results are similar with the band structures of other defective oxides such as ZnO and Al_2_O_3_^30^,^33^.

**Figure 5:**
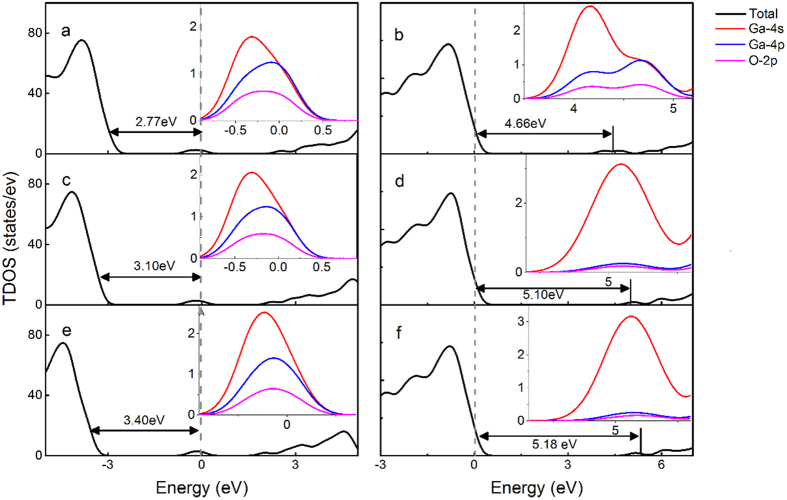
DOS of defects in (**a**) 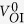, (**b**)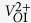, (**c**) 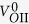, (**d**)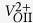, (**e**)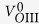, and (**f**) 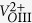 states, the inset figure is the PDOS caused by the defects.

**Figure 6:**
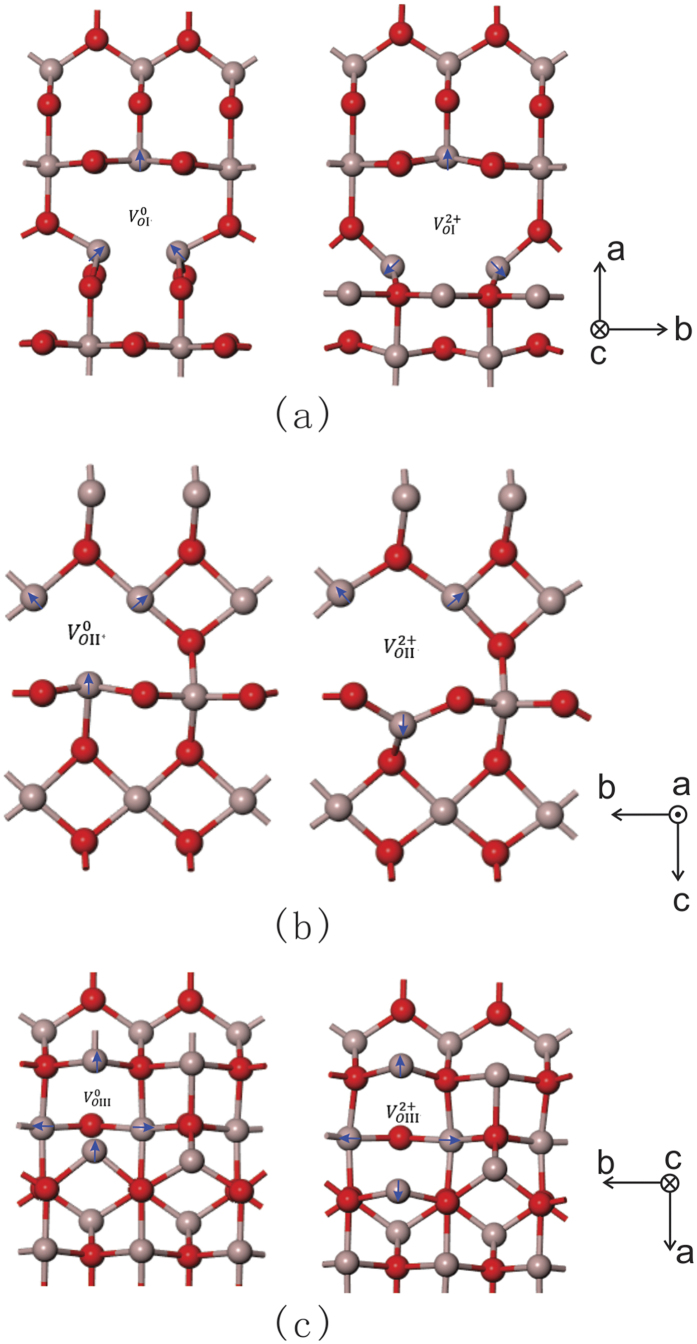
The atomic structures relaxations of (**a**) 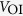, (**b**) 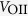, and (**c**) 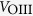.

Figure 5 presents the total density of states (TDOS) of β-Ga_2_O_3_ with various oxygen vacancies, and detailed partial density of states (PDOS) results induced by the defects are shown inset. Compared with the TDOS of the intrinsic β-Ga_2_O_3_, new peaks arise in the bandgap. For 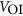, with the VBM chosen as the reference, the DOS peak of 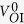 is 2.77 eV away from the VBM. Based on the PDOS of the Ga ions and O ions shown in Fig. 5(a,b), the unpaired hanging bond of Ga ions around the vacancies make the most contributions to these defective peaks. The DOS of defects almost come from Ga-4s and Ga-4p states along with a few O-2p states. In the presence of 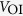 shown in Fig. 2(a), it is found that Ga1 and Ga3 ions move 0.21 Å toward the vacancy, while Ga2 slightly move about 0.16 Å away from the oxygen vacancy, which is shown in Fig. 6(a). The movement of the Ga ions can be attributed to the ionic size and crystalline structure. When two electrons move away, the defects level caused by the charged oxygen vacancies move toward the conduction band, the interaction caused by the overlap between the defects level and the conduction bottom level make the conduction band bottom shift down, as shown in Fig. 4(d). The attractive interactions between Ga ions and O vacancy disappear. There will be considerate space left in the vacancy site, which will give the adjacent Ga ions more freedom to disperse. For both equivalent tetrahedral structure Ga1 and Ga3 ions, the space makes them move toward to vacancy after the relaxation. While for Ga2 ion, Ga2 ion is not electrostatically attracted by the vacancy anymore, the closer Ga1 and Ga3 ions product more electrostatically repulsion to Ga2 ion, these effects leave Ga3 ions move away. However, compared with Ga1 and Ga3 ions, Ga2 ion with an octahedral structure has seven bonds connected to neighboring oxygen ions, which make Ga2 ion more geometrically stable.

Similar to 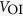, the band structures and DOS of 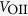 and 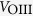 shown in Figs 4 and 5, respectively. Extra levels emerge in the band structures, with the VBM chosen as the reference, the DOS peak of 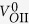 and 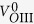 is 3.10 eV and 3.40 eV away from the VBM, respectively. The structural relaxations of the supercell models with 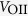 and 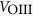 are also calculated, and the variation between the defect and surrounding ions after relaxation are listed in Table 2. Detailed atomic structures relaxations shown in Fig. 6(b,c). It is noted that the 4-fold 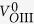 connected there octahedron and one tetrahedron, which makes the 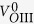 more geometrically stable. As a result, when 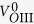 induced, the variation of adjacent Ga ions are less, the three equivalent 6-fold Ga ions move less away from the 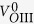, more relaxation is applied to 4-fold Ga1 ion, the result is Ga1 ion is attracted to the vacancy 0.33 Å closer.

### Optical properties

As a promising material for optoelectronic devices, a deep understanding of optical properties of β-Ga_2_O_3_ is very necessary. To investigate the optical properties of β-Ga_2_O_3_ with oxygen vacancies, the complex dielectric function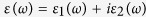 is calculated. The imaginary part of complex dielectric function *ε*_2_(*ω*) is calculated by summing the transitions between occupied and unoccupied electronic states, which is relevant to the electronic band structure that can determine other optical properties of the material. The imaginary part is given by the following equation^34^:


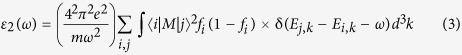


Where *e* is the electron charge, *m* is the mass of free electrons, *ω* is the frequency of incident photons, *M* is the dipole matrix, *i* and *j* are the initial and final states, respectively. *f*_*i*_ is the fermi distribution function for *i*-th state with wave function vector *k*. According to the Kramers-Kronig transformation, the real part *ε*_1_ can derived from the imaginary *ε*_2_, which is given as follows:


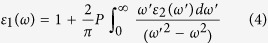


Where *P* denotes the principal value of the integral. The other optical properties such as absorption coefficient *α*(ω), reflectivity *R*(ω), refractive index *n*(ω), extinction coefficient *k*(ω), and energy-loss *L*(ω) can be derived from the dielectric function and defined by^35^:


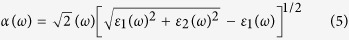



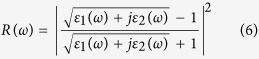



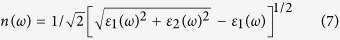



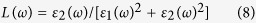


**Figure 7:**
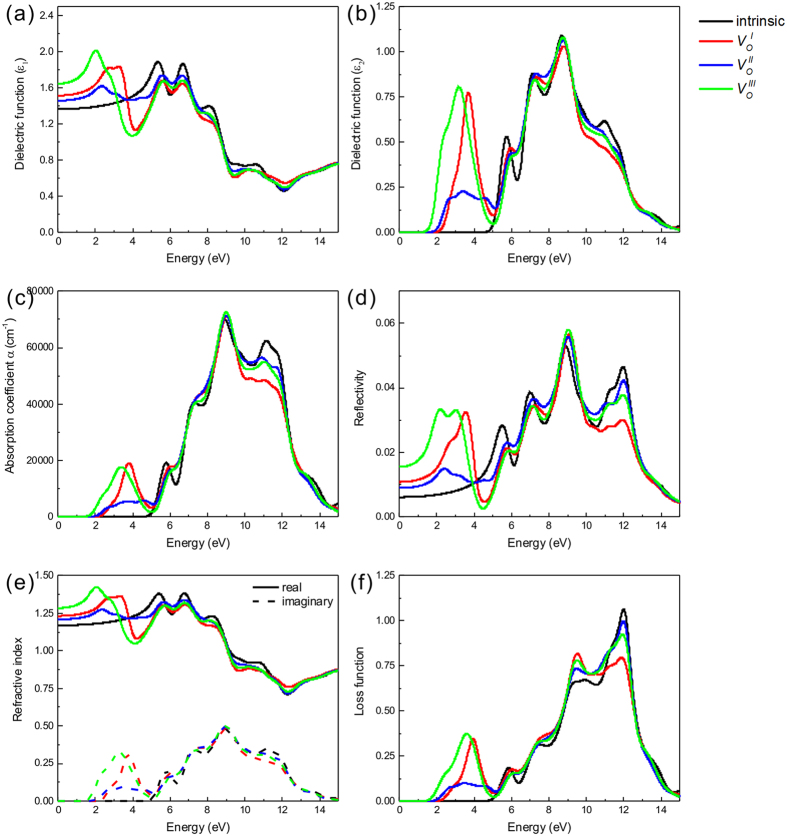
The real part (**a**), the imagine part (**b**) of the dielectric function, absorption coefficient (**c**), reflectivity (**d**), refractive index (**e**), and electron-loss function (**f**) of β-Ga_2_O_3_ with oxygen vacancies.

Figure 7(a,b) display the real and imaginary parts of the dielectric function *ε*(ω) of β-Ga_2_O_3_ with different oxygen vacancies, respectively. For the pure β-Ga_2_O_3_, from a general view, our calculation dielectric function *ε*(ω) are consistent with previous studies in tendency. It is noted that the peak for the imaginary part dielectric function at 8.7 eV is much stronger than other peaks, which is related to the interband transition between O 2p and Ga 4 s states. Taking the oxygen vacancies into consideration, new peaks (3.17eV, 3.37eV and 3.69 eV for 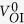, 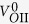 and 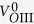, respectively) arise in the low energy region, while the effect is barely noticeable in ultraviolet region. From the electronic structures and DOS results, we concluded that these peaks are caused by the transition from Ga-4s states in the defective level induced by oxygen vacancies to the Ga-4s states in the conduction band. The difference locations among these peaks are consistent with the defective levels in the band structures from our previous calculated results.

The static dielectric constant *ε*_1_(0) is given by the low energy limit of *ε*_1_(∞). The calculated *ε*_1_(0) of pure β-Ga_2_O_3_ is 1.36, which is smaller than the experiment results. The underestimation of *ε*_1_(0) is due to the low number of conduction bands and overlook of phonon contribution. When oxygen vacancies induced in the β-Ga_2_O_3_, all the values of *ε*_1_(0) increase, the *ε*_1_(0) for β-Ga_2_O_3_ with 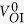, 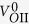 and 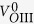 are 1.52, 1.46 and 1.64, respectively, which are attributed to the extra levels in the bandgap of β-Ga_2_O_3_ caused by oxygen vacancies.

**Figure 8:**
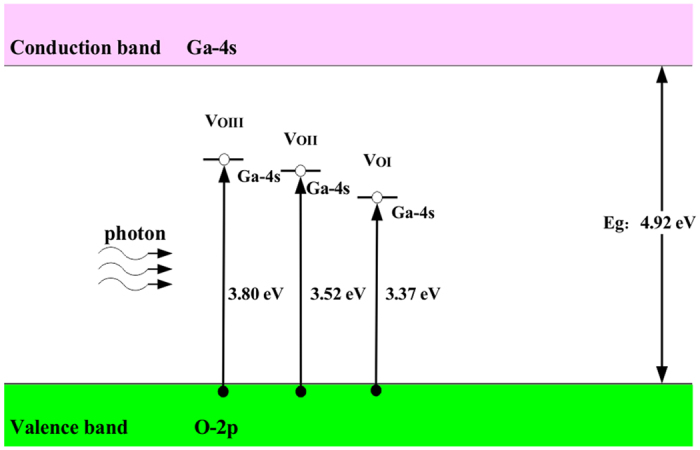
The possible absorption processes of oxygen vacancies in β-Ga_2_O_3_. Filled circles denote electrons and empty circles denote holes.

The absorption spectra of all the β-Ga_2_O_3_ systems are illustrated in Fig. 7(c). The incident radiation has linear polarization along the (100) direction. The intrinsic absorption edge of β-Ga_2_O_3_ is consistent with the bandgap (4.9 eV) with the urgently decreases absorption edge, which means β-Ga_2_O_3_ is a promising optical material at DUV region. The intrinsic absorption is related with the interband electron transition between O-2p states in the VBM and Ga-4s states in the CBM. Compared with pure β-Ga_2_O_3_, the absorption coefficient of the structures with oxygen vacancies increase in visible and infrared region while decrease in the deep violet region. New absorption peaks appear at 3.80 eV, 3.52 eV and 3.37 eV for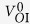, 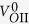 and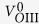, respectively. According our first-principles calculation results, a schematic diagram of the possible absorption processes of oxygen vacancies in β-Ga_2_O_3_ is illustrated in Fig. 8. The intrinsic absorption process is the electron transition from O-2p states in the VBM and Ga-4s states in the CBM. The additional absorption parts are deduced from the electron transition between the defective level caused by oxygen vacancies and the valence band.

The reflectivity, and refractive index are depicted in Fig. 7(d,e). For β-Ga_2_O_3_ with oxygen vacancies, the reflectivity and refractive index enhance in the low energy region, which is contributed to the defective levels in the bandgap induced by the oxygen vacancies. The location of reflectivity and refractive index peaks are consistent with the dielectric function. The energy loss function describes the energy lost by an electron passing from a homogeneous dielectric material. It has the advantage of covering the complete energy range, including non-scattered and elastically scattered electrons (zero loss), which excite the electrons of atom’s outer shell (valence loss) or valence inter-band transitions. In Fig. 7(f), the energy loss peaks caused by oxygen vacancies locates at 3.96eV, 3.45eV and 3.58 eV for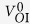, 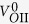 and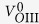, respectively. These peaks are corresponding to where the reflectivity decrease rapidly.

### XRD and photoluminescence spectra results

**Figure 9:**
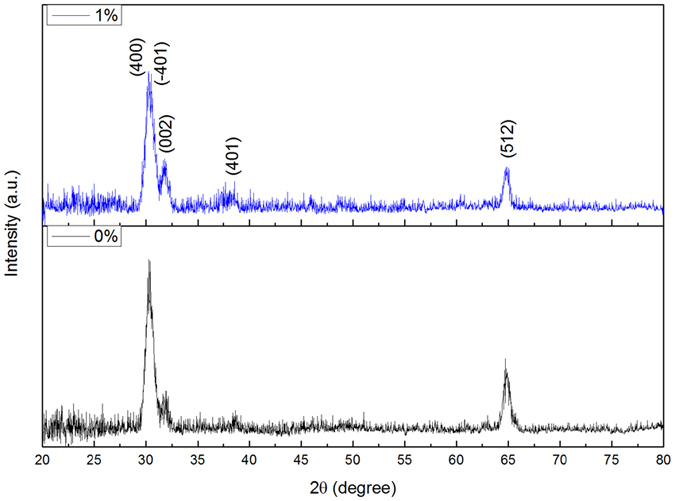
XRD patterns of the β-Ga_2_O_3_ films deposited under different O_2_ volume percentage.

**Figure 10:**
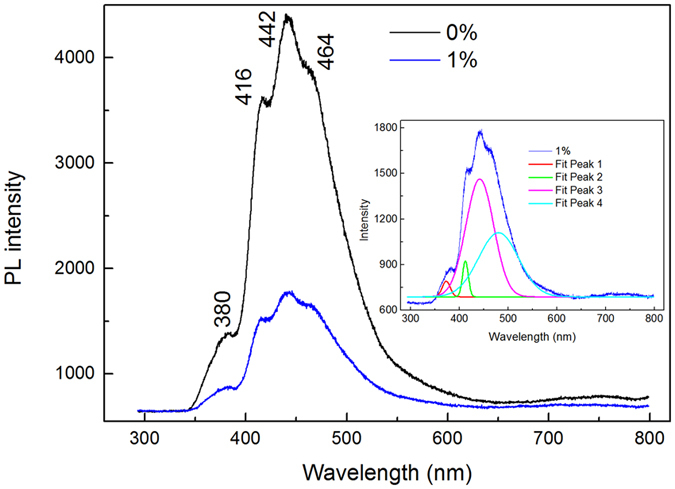
PL spectra of the β-Ga_2_O_3_ films deposited under different O_2_ volume percentage.

To explore the effects of oxygen vacancies on the structural and optical properties of β-Ga_2_O_3_, β-Ga_2_O_3_ films were deposited under different O_2_ volume percentage. Figure 9 presents the XRD results of the films deposited under 0% and 1% O_2_ volume percentage, respectively. Through XRD patterns, both films exhibit β-Ga_2_O_3_ structure. The average crystalline sizes in β-Ga_2_O_3_ films were estimated from the (400) peak by using the Scherrer’s equation, D = kλ/(B × cosθ), where k is Scherrer’s constant with a value of 0.89, λ is the wavelength of the X-ray radiation, B is the FWHM of (400) peak, and θ is the angle of the diffraction peak. The calculated crystalline sizes for the films deposited under 0% and 1% O_2_ volume percentage are 20.15 nm and 22.45 nm, respectively. Room-temperature photoluminescence (PL) spectra of the β-Ga_2_O_3_ films excited with 325 nm laser are shown in Fig. 10. The fitting curve of the film deposited under 1% O_2_ volume percentage is shown in the inset figure. The emission band can be divided into four Gaussian bands centered at about 380 nm, 416 nm, 442 nm and 464 nm, respectively. The emission peak centers at 380 is in the UV region, this peak is concerned with the transition levels between the oxygen vacancy and unintended N impurities introduced in the N_2_ annealed^36^. While for the peaks center at 416 nm, 442 nm (both in the violet region) and 464 nm (blue region), these three emission peaks are originated from the electron-hole recombination formed by oxygen vacancies, or to the recombination of Ga-O vacancy pair^37^,^38^.

From our previous calculated results, extra oxygen gas induced in the procedure of deposition can increase the formation energy of oxygen vacancies. The higher formation energy can reduce the concentration of oxygen vacancies. From the PL results, the emission peaks related to oxygen vacancies of the film deposited under 1% O_2_ volume percentage is higher than that deposited under 0%. According to our first-principles calculation results, the peaks center at 416 nm and 442 nm are most related with 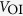, and 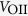, while the peak at 464 nm is most attributed to 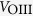. When irradiated with high energy photon, the electron at defective levels caused by oxygen vacancies can absorb the photon and then jump to the levels in the valance band consists of O-2p states or the defective level caused by Ga vacancies, with more photons release in this procedure.

In summary, we have investigated the structural, electronic and optical properties of defected β-Ga_2_O_3_ with oxygen vacancies by employing first-principles calculations based on density function theory. The relaxed lattice parameters are in consistent with the experiment and previous theoretical results. The variation in defective structures caused by the O vacancies is discussed systematically. To revise the error calculated under the Generalized Gradient Approximation, the electronic structures, and optical properties of β-Ga_2_O_3_ are calculated by GGA+U method. Our calculated bandgap result (4.92 eV) is in good agreement with the previous experiment results. The formation energies of the oxygen vacancies and transition levels between different charge states are calculated, the results indicate that +2 charge states are most stable state while +1 charge states are not stable for all three type O vacancies, the +2/0 transition level location implying O vacancy is a deep donor. When the ambient atmosphere becomes oxygen poor, the oxygen vacancies are easier to form. The electronic structures show the oxygen vacancies can introduce deep levels around the Fermi level, detailed PDOS of the defects level are described. The static real dielectric constants of the defective structures increase compared with the intrinsic one, which is attributed to the level caused by the Ga-4s states in the bandgap. New absorption peaks appear at 3.80 eV, 3.52 eV and 3.37 eV for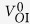, 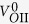 and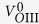, respectively. The additional absorption parts are deduced from the electron transition between the defective level caused by oxygen vacancies and the valence band. The absorption intensity is increasing in visible and infrared region but decreasing in the deep violet region. Besides, β-Ga_2_O_3_ films are deposited under different O_2_ volume percentage with ratio frequency magnetron sputtering method. The measured results indicate that oxygen vacancies can induce extra emission peaks in the photoluminescence spectrum of β-Ga_2_O_3_, the location of these peaks is close to our calculated results. Extra O_2_ can increase the formation energies of oxygen vacancies and reduce oxygen vacancies in β-Ga_2_O_3_. These results are consistent with first-principles calculations results.

## Methods

All calculations were based on the density functional theory with Cambridge Serial Total Energy Package (CASTEP) code^39^. The exchange-correlation potential was described with the Perdew-Burke-Ernzerhof (PBE) functional under the Generalized Gradient Approximation (GGA) exchange-correlation functional^40^. The ultrasoft pseudopotential method was used for the interactions between the electrons and ions. The atomic configuration of Ga was [Ar] 3*d*^10^, and the 3*d*^10^ electrons were considered as valence electrons, while the atomic configuration of O is [He] 2*s*^2^2*p*^4^, the 2*s*^2^ and 2*p*^4^ electrons were treated as valence electrons. Before the electronic and optical calculations, structural relaxation was employed. The lattice parameters and internal coordinates were relaxed with Broyden-Fletcher-Goldfarb-Shanno (BFGS) optimization method^41^. The energy tolerance, the tolerance of the force, maximum stress and maximum displacement were 5 × 10^−6^ eV/atom, 0.01 eV/Å, 0.02 Gpa and 5 × 10^−4^ Å, respectively. The cutoff energy for the plane wave basis set was 450 eV, and a Monkhorst-Pack 2 × 8 × 4 k-points was used for integrations of the Reduced Brillouin zone^42^. For the defective crystal, a 1 × 2 × 2 supercell of β-Ga_2_O_3_ based on the optimized cell with 80 atoms was created to act as the computational model, which is presented in Fig.1 (b). The lattice constants of the defective supercell were fixed, only the internal coordinates can be relaxed.

For the calculations of formation energies, electronic structures and optical properties, the GGA+U method was adopted. After series tests, the U_d_ value for Ga-3d and U_p_ value for O-2p were set at 7.0 eV and 8.5 eV, respectively. Under this correction, the reasonable results can be obtained. All of our calculations are carried out at the theoretical equilibrium lattice constants, which is essential in order to avoid the spurious effects in the process of relaxation.

β-Ga_2_O_3_ films were prepared on sapphire substrates (0001) by the radio-frequency magnetron sputtering method with a high-purity Ga_2_O_3_ target (99.995% purity, 50.8 mm diameter). The distance between the target and substrate was about 150 mm, and the output of the ratio source was 60 W. To explore the oxygen vacancies on the properties of β-Ga_2_O_3_ films, several experiments were conducted by varying the oxygen concentration in the growth chamber. Before the deposition, the chamber was pumped down to 5 × 10^−6^ mTorr as the base pressure. The films were deposited under 5 mTorr atmosphere pressure at room temperature. To exclude any interference caused by the variation of film thickness, the thickness of both films were kept between 220 to 250 nm. To improve the crystalline properties, the as-deposited Ga_2_O_3_ sample was subsequently annealed at 1000 °C for 60 min. Pure N_2_ was chosen as the annealing atmosphere to avoid any effects of caused by extra oxygen. The oxygen volume percentage was varied by changing the gas volume percentage of oxygen to argon gas introduced into the growth chamber. To investigate the crystalline structures of the films, 2θ scan were conducted using an X-ray diffractometer (SHIMADZU XRD-7000), with Cu Kα radiation (λ = 0.154056 nm). The Photoluminescence (PL) spectra were measured under the excitation by a He-Cd UV laser (325 nm and 20 mW).

## Additional Information

**How to cite this article**: Dong, L. *et al*. Effects of oxygen vacancies on the structural and optical properties of β-Ga_2_O_3_. *Sci. Rep.* **7**, 40160; doi: 10.1038/srep40160 (2017).

**Publisher's note:** Springer Nature remains neutral with regard to jurisdictional claims in published maps and institutional affiliations.

## Figures and Tables

**Table 1:** Calculated structural parameters of β-Ga_2_O_3_ and the previous theoretical along with experimental results.

β-Ga_2_O_3_	Functional	a(Å)	b(Å)	c(Å)	β(°)	Formation energy (eV)
This work	GGA	12.55	3.08	5.89	103.67	−9.57
Ref. 13	GGA	12.45	3.08	5.88	103.70	−9.3
Ref. 14	HSE06	12.27	3.05	5.82	103.82	—
Ref. 18	B3LYP	12.34	3.08	5.87	103.9	—
Exp^29^	—	12.23	3.04	5.80	103.7	−11.3

**Table 2:** Structural relaxations around each oxygen vacancy and average distances from the defect positions to neighboring ions.

Vacancy type	Distance in Å (the Ga coordination number around O vacancy which is shown in Fig. 1 (b), +denotes Ga ions move close, while −denotes Ga ions move away)			
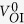	0.21(Ga1, +)	0.21(Ga3, +)	0.16(Ga2, −)	—
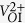	0.38(Ga1, −)	0.38(Ga3, −)	0.29(Ga2, −)	—
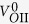	0.03(Ga4, −)	0.03(Ga5, −)	0.34(Ga1, +)	—
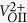	0.27(Ga4, −)	0.27(Ga5, −)	0.85(Ga1, −)	—
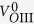	0.06(Ga6, −)	0.06(Ga7, −)	0.33(Ga1, +)	0.06(Ga8, −)
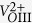	0.21(Ga6, −)	0.21(Ga7, −)	0.96(Ga1, −)	0.21(Ga8, −)

## References

[b1] Orita M., Ohta H., Hirano M. & Hosono H. Deep-ultraviolet transparent conductive beta-Ga_2_O_3_ thin films. Appl Phys Lett 77, 4166 (2000).

[b2] Kumar S., Tessarek C., Sarau G., Christiansen S. & Singh R. Self‐Catalytic Growth of β‐Ga_2_O_3_ Nanostructures by Chemical Vapor Deposition. Adv Eng Mater 17, 709–715 (2015).

[b3] Víllora E. G., Arjoca S., Shimamura K., Inomata D. & Aoki K. In SPIE OPTO. (International Society for Optics and Photonics), pp. 89871U-89871U–89812 (2014).

[b4] Jin S. Q. . Effect of Phase Junction Structure on the Photocatalytic Performance in Overall Water Splitting: Ga_2_O_3_ Photocatalyst as an Example. J Phys Chem C 119, 18221–18228 (2015).

[b5] Suzuki R., Nakagomi S., Kokubun Y., Arai N. & Ohira S. Enhancement of responsivity in solar-blind β-Ga_2_O_3_ photodiodes with a Au Schottky contact fabricated on single crystal substrates by annealing. Appl. Phys. Lett 94, 222102–222101 (2009).

[b6] Chen X. . Self-Powered Solar-Blind Photodetector with Fast Response Based on Au/beta-Ga_2_O_3_ Nanowires Array Film Schottky Junction. ACS Appl Mater Interfaces 8, 4185–4191 (2016). 26817408 10.1021/acsami.5b11956

[b7] Ogita M., Higo K., Nakanishi Y. & Hatanaka Y. Ga_2_O_3_ thin film for oxygen sensor at high temperature. Appl Surf Sci 175, 721–725 (2001).

[b8] Matsuzaki K. . Field-induced current modulation in epitaxial film of deep-ultraviolet transparent oxide semiconductor Ga_2_O_3_. Appl Phys Lett 88 (2006).

[b9] Higashiwaki M. . Depletion-mode Ga_2_O_3_ metal-oxide-semiconductor field-effect transistors on β-Ga_2_O_3_ (010) substrates and temperature dependence of their device characteristics. Appl Phys Lett 103, 123511 (2013).

[b10] Higashiwaki M., Sasaki K., Kuramata A., Masui T. & Yamakoshi S., Development of gallium oxide power devices. physica status solidi (a) 211, 21–26 (2014).

[b11] Higashiwaki M., Sasaki K., Kuramata A., Masui T. & Yamakoshi S., Gallium oxide (Ga_2_O_3_) metal-semiconductor field-effect transistors on single-crystal beta-Ga_2_O_3_ (010) substrates. Appl Phys Lett 100 (2012).

[b12] Hajnal Z. . Role of oxygen vacancy defect states in the n-type conduction of beta-Ga_2_O_3_. J Appl Phys 86, 3792–3796 (1999).

[b13] Zacherle T., Schmidt P. C. & Martin M. Ab initio calculations on the defect structure of beta-Ga_2_O_3_. Phys Rev B 87 (2013).

[b14] Varley J. B., Weber J. R., Janotti A. & Van de Walle C. G. Oxygen vacancies and donor impurities in beta-Ga_2_O_3_. Appl Phys Lett 97 (2010).

[b15] Guo D. Y. . Abnormal bipolar resistive switching behavior in a Pt/GaO1.3/Pt structure. Appl Phys Lett 107, 032104 (2015).

[b16] Hsu C. W. & Chou L. J. Bipolar resistive switching of single gold-in-Ga_2_O_3_ nanowire. Nano Lett 12, 4247–4253 (2012). 22823742 10.1021/nl301855u

[b17] Lee D. Y. & Tseng T. Y. Forming-free resistive switching behaviors in Cr-embedded Ga_2_O_3_ thin film memories. J Appl Phys 110 (2011).

[b18] He H. Y. . First-principles study of the structural, electronic, and optical properties of Ga_2_O_3_ in its monoclinic and hexagonal phases. Phys Rev B 74 (2006).

[b19] Di Trolio A. . The effect of Co doping on the conductive properties of ferromagnetic ZnxCo1−xO films. J. Mater. Chem. C 3, 10188–10194 (2015).

[b20] Zhou R., Qu B., Zhang B., Li P. & Zeng X. C. Role of vacancies to p-type semiconducting properties of SiGe nanowires. J Mater Chem C 2, 6536 (2014).

[b21] Wang Y. . Zn vacancy induced ferromagnetism in K doped ZnO. J. Mater. Chem. C 3, 11953–11958 (2015).

[b22] Perdew J. P. & Zunger A. Self-interaction correction to density-functional approximations for many-electron systems. Phys Rev B 23, 5048 (1981).

[b23] Ceperley D. M. & Alder B. Ground state of the electron gas by a stochastic method. Phys Rev Lett 45, 566 (1980).

[b24] Perdew J. P., Kurth S., Zupan A. & Blaha P. Accurate density functional with correct formal properties: A step beyond the generalized gradient approximation. Phys Rev Lett 82, 2544 (1999).

[b25] Rinke P., Janotti A., Scheffler M. & Van de Walle C. G. Defect formation energies without the band-gap problem: combining density-functional theory and the GW approach for the silicon self-interstitial. Phys Rev Lett 102, 026402 (2009). 19257298 10.1103/PhysRevLett.102.026402

[b26] Liu D., Guo Y., Lin L. & Robertson J. First-principles calculations of the electronic structure and defects of Al_2_O_3_. J Appl Phys 114, 083704 (2013).

[b27] Liechtenstein A., Anisimov V. & Zaanen J. Density-functional theory and strong interactions: Orbital ordering in Mott-Hubbard insulators. Phys Rev B 52, R5467 (1995). 10.1103/physrevb.52.r5467 9981806

[b28] Dudarev S., Botton G., Savrasov S., Humphreys C. & Sutton A. Electron-energy-loss spectra and the structural stability of nickel oxide: An LSDA+ U study. Phys Rev B 57, 1505 (1998).

[b29] Geller S. Crystal Structure of β‐Ga_2_O_3_. The Journal of Chemical Physics 33, 676–684 (1960).

[b30] Matsunaga K., Tanaka T., Yamamoto T. & Ikuhara Y. First-principles calculations of intrinsic defects inAl_2_O_3_. Phys Rev B 68 (2003).

[b31] Mattila T. & Zunger A. Deep electronic gap levels induced by isovalent P and As impurities in GaN. Phys Rev B 58, 1367 (1998).

[b32] Herzberg G. Forbidden Transitions in Diatomic Molecules: I. The Quadrupole Rotation-Vibration Spectrum of H2. Canadian Journal of Research 28, 144–152 (1950).

[b33] Oba F., Togo A., Tanaka I., Paier J. & Kresse G., Defect energetics in ZnO: A hybrid Hartree-Fock density functional study. Phys Rev B 77, 245202 (2008).

[b34] Chen C. . Nonlinear Optical Borate Crystals: Principals and Applications. (John Wiley & Sons, 2012).

[b35] Li L. . First principles calculations of electronic band structure and optical properties of Cr-doped ZnO. The Journal of Physical Chemistry C 113, 8460–8464 (2009).

[b36] Liu L. . Fabrication and characteristics of N-doped β-Ga_2_O_3_ nanowires. Applied Physics A 98, 831–835 (2010).

[b37] Binet L. & Gourier D. Origin of the blue luminescence of β-Ga_2_O_3_. J Phys Chem Solids 59, 1241–1249 (1998).

[b38] Chang K. W. & Wu J. J. Low‐Temperature Growth of Well‐Aligned β‐Ga_2_O_3_ Nanowires from a Single‐Source Organometallic Precursor. Adv Mater 16, 545–549 (2004).

[b39] Clark S. J. . First principles methods using CASTEP. Zeitschrift für Kristallographie-Crystalline Materials 220, 567–570 (2005).

[b40] Perdew J. P., Burke K. & Ernzerhof M. Generalized gradient approximation made simple. Phys Rev Lett 77, 3865 (1996). 10062328 10.1103/PhysRevLett.77.3865

[b41] Pfrommer B. G., Côté M., Louie S. G. & Cohen M. L. Relaxation of crystals with the quasi-Newton method. Journal of Computational Physics 131, 233–240 (1997).

[b42] Monkhorst H. J. & Pack J. D. Special points for Brillouin-zone integrations. Phys Rev B 13, 5188 (1976).

